# An Investigation into the Interaction between Double Hydroxide-Based Antioxidant Benzophenone Derivatives and Cyclooxygenase 2

**DOI:** 10.3390/molecules26216622

**Published:** 2021-11-01

**Authors:** Yanan Qiao, Yuxi Qin, Lihua Liu, Xi Chen, Yunlan Li, Qingshan Li

**Affiliations:** 1 School of Pharmaceutical Science, Shanxi Medical University, Taiyuan 030001, China; yananqiao@sxmu.edu.cn (Y.Q.); qinyvxi@163.com (Y.Q.); llh9707@163.com (L.L.); anne_chenxi@163.com (X.C.); 2 School of Public Health, Shaanxi University of Chinese Medicine, Xi’an 712046, China; 3 Key Laboratory of Innovative Drug for the Treatment of Serious Diseases Basing on the Chronic Inflammation, Shanxi University of Chinese Medicine, Taiyuan 030001, China

**Keywords:** double hydroxide-based benzophenone derivatives, cyclooxygenases 2, interaction, biolayer interferometry, molecular docking, multi-spectroscopy studies, antioxidant

## Abstract

Cyclooxygenases 2 (COX2) is a therapeutic target for many inflammation and oxidative stress associated diseases. A high-throughput technique, biolayer interferometry, was performed to primarily screen the potential COX2 binding activities of twelve newly synthesized double hydroxide-based benzophenone derivatives. Binding confirmation was achieved by molecular docking and multi-spectroscopy studies. Such a combined method provided a comprehensive understanding of binding mechanism and conformational changes. Compounds DB2, SC2 and YB2 showed effective COX2 binding activity and underlined the benefits of three phenolic hydroxyl groups adjacent to each other on the B ring. The twelve tested derivatives were further evaluated for antioxidant activity, wherein compound SC2 showed the highest activity. Its concentration for the 50% of maximal effect (EC_50_) value was approximately 1000 times greater than that of the positive controls. SC2 treatment effectively improved biochemical indicators caused by oxidative stress. Overall, compound SC2 could serve as a promising candidate for further development of a new potent COX2 inhibitor.

## 1. Introduction

Cyclooxygenases (COXs) are enzymes that catalyze the rate-limiting enzyme in the production of the bioactive molecules termed prostanoids [1,2,3]. The inducible form of cyclooxygenases, COX2, is expressed in response to inflammatory stimuli such as cytokines and bacterial endotoxin. The pathophysiological importance of COX2 renders the enzyme an appealing target for the treatment of many inflammation and oxidative stress associated diseases like chronic pain, rheumatoid arthritis and cancer [3,4]. Therefore, strong emphasis is placed on developing analgesics that interact with COX2 and inhibit its enzymatic activity [5,6,7]. Nonsteroidal anti-inflammatory drugs (NSAIDs) are the classical COX inhibitors and widely prescribed [3]. Nevertheless, these medications have shown to induce severe adverse effects, such as gastrointestinal bleeding and renal disorders [2]. These serious complications associated with NSAIDs restrict the clinical application, especially in long-term therapy. Some NSAIDs were withdrawn from the market due to cardiovascular events [8]. Therefore, there is still an urgent need for new COX2 inhibitors with an improved safety profile. 

**Figure 1:**
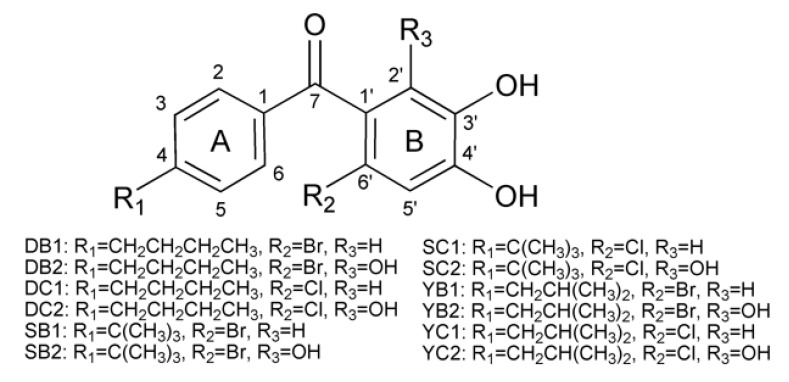
Structures of twelve polyhydroxy benzophenone derivatives.

Double hydroxide benzene derivatives are substances with more than one phenolic hydroxyl group from synthetic or natural origin. They present a variety of pharmacological properties, including antiviral [9], antimicrobial [10,11], free radical-scavenging [12], anti-inflammatory [13], anticancer [14], antiproliferative [15], management of obesity [16] and Parkinson’s disease [17,18]. Among these properties, anti-inflammatory and antioxidant activities are highlighted. Because extensive oxidative stress and inflammation contribute to the occurrence of molecular and cellular damage directly or indirectly, there is an increased risk for other complications, including sclerosis as well as heart, respiratory and neurodegenerative diseases [19,20]. It is important to mention that dihydroxybenzenes exhibit a wide spectrum of anti-inflammatory and antioxidant activities owing to the free radicals blocking ability of phenolic hydroxyl groups [12,21]. Earlier, we have reported a series of patented compounds based on double hydroxide benzophenone (Chinese patent number 201710278835.7, ZL201610278158.4 and 202010778813.5, Japanese patent number 6698214) showing anti-inflammatory activity [22,23]. We found that the presence of chloric or bromo atoms (electron withdrawing groups) in the ortho-position increased the in vitro anti-inflammatory activities significantly, which was consistent with previous studies [24,25,26]. The same applied to the introduction of a butyl group in the para-position of the A ring. To develop improved compounds with higher COX2 binding affinity based on our previous studies, recently, we designed and synthesized twelve analogs of double hydroxide-based benzophenone by maintaining the main pharmacophore and introducing a hydroxyl group in the ortho-position of the B ring (Figure 1).

In this study, the interactions between the twelve compounds and COX2 were investigated. Firstly, a high-throughput and efficient screening technique was necessary to quickly and accurately determine whether the selected small molecules can bind to the protein molecule. Recently, a relatively new method, biolayer interferometry (BLI) is increasingly being utilized for the real-time assay of molecular interactions between an immobilized ligand and targets [27,28]. It is based on disposable optical fiber sensors. It can provide binding profiles and specific kinetic parameters directly [29]. The BLI experiments are foundational for all subsequent experiments. Only by confirming the binding between small molecules and COX2, can the subsequent mechanism and conformation studies be meaningful. Molecular docking can be applied alongside experimental techniques to further explore the binding mode. In addition, multi-spectral studies can be used to confirm the binding of the prescreened compounds with COX2 and to further clarify the binding characteristic. To the best of our knowledge, our experiments are the first example to combine BLI, molecular docking and multi-spectral technologies to study the conformational changes and interaction mechanism between double hydroxide-based benzophenone derivatives and COX2. Compared with the sole experimental results, this combination of integrated computational simulation and experimental results is more explicit and intuitive. It is also more convincing than the pure theoretical study. 

Inflammation and oxidative stress are inextricably intertwined. Both processes can trigger and promote one another [30,31]. Finally, twelve novel compounds were tested for their antioxidant activity against H_2_O_2_-induced cell damage. The most promising candidate, SC2, was also evaluated for the activities to improve biochemical indicators caused by oxidative stress.

## 2. Results

### 2.1. Biolayer Interferometry Studies

**Figure 2:**
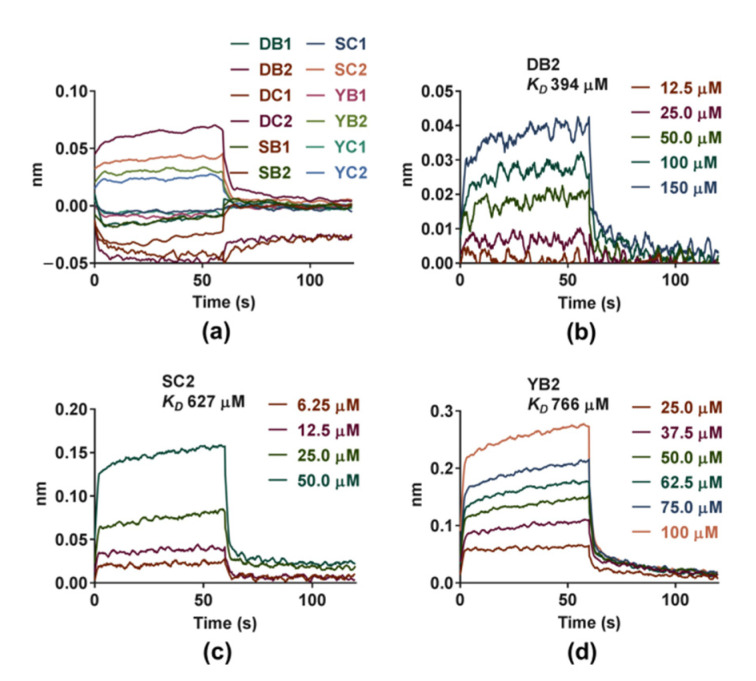
Sensograms for the interaction of 12 compounds with cyclooxygenases 2 (COX2) obtained with biolayer interferometry (BLI); (**a**) All 12 compounds were initially assayed at a single concentration of 100 μM. (**b**–**d**) The results showed a medium-high affinity of compounds DB2 (**b**), SC2 (**c**) and YB2 (**d**) to COX2.

The label-free real-time binding assay, BLI, was firstly performed to evaluate the potential COX2 binding activities of the twelve novel compounds. COX2 protein was captured onto the Ni-NTA sensors, by the virtue of its His-tag, followed by exposure to small-molecule compound. The binding of small-molecule compound to the biosensor increased the layer thickness of the sensor, resulting in an interference wavelength shift recorded by BLI. All 12 compounds were initially assayed at a single concentration of 100 μM (Figure 2a). Compounds showed binding to COX2 protein in the single concentration test were re-assayed using a 4–6 point concentration series. 

A significant shift in the association curve was observed upon addition of different concentrations of compounds DB2, SC2 and YB2 to COX2 in a concentration dependent manner (Figure 2b–d). During the dissociation step, it was also observed that the signal decreased to the baseline. That was a sign of a total protein-ligand dissociation, thus indicating the interaction was reversible. The real-time binding curves were generated fitting to a one-to-one binding model. The ratio of *k*_*off*_ (dissociation) and *k*_*on*_ (association) constants was equilibrium dissociation constants (*K_D_*). Results showed a medium-high affinity [32] of compounds DB2, SC2 and YB2 to COX2 with a *K_D_* value of 394 μM, 627 μM and 766 μM, respectively.

### 2.2. Molecular Docking Studies

Molecular docking is the assay process of the geometric and capacity matching of molecules. It is usually used to predict drug–target binding mode. 

**Figure 3:**
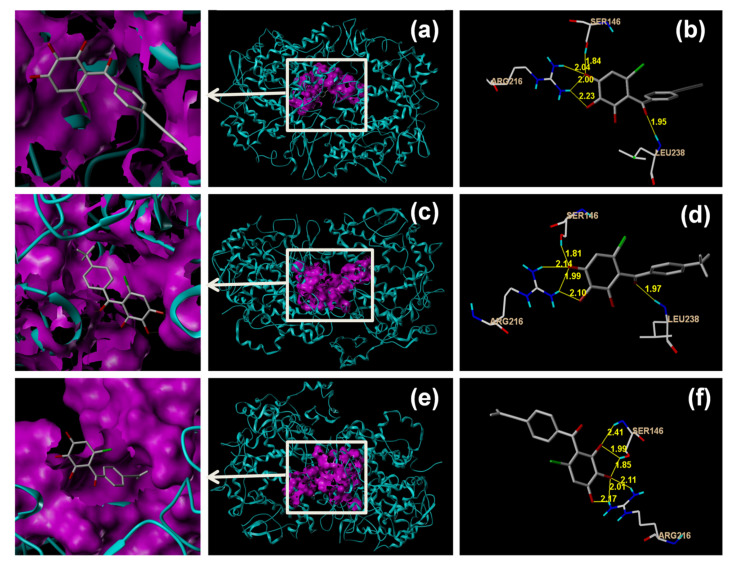
3D putative binding complexes and hydrogen bonding of compounds DB2 (**a**,**b**), SC2 (**c**,**d**) and YB2 (**e**,**f**) with the COX2 enzyme (PDB code 5f1a). (COX2 is shown as cyan ribbon; The ligand surface is indicated in purple; Compounds DB2, SC2 and YB2 are shown in capped sticks models; The amino acids involved in hydrogen bond contact are shown in line models; Hydrogen bonding are yellow lines).

Figure 3a,b, Figure 3c,d, and Figure 3e,f showed the binding of compounds DB2, SC2 and YB2 against COX2 enzyme, respectively. Figure 3c showed that in the interaction between compound SC2 and COX2, compound SC2 was connected to the protein active pocket (purple area) through hydrogen bonds. Figure 3d showed that the compound bound to the active pocket of COX2 through five hydrogen bonds. Four hydrogen bonds connected the phenolic hydroxyl groups of compound SC2 and the amino acids Ser146 (bond length 1.81 Å) and Arg216 (bond length 1.99 to 2.14 Å) of COX2. In addition, the amino acid residue Leu238 formed one hydrogen bond with the carbonyl group of compound SC2 (bond length 1.97 Å).

Compound DB2 was placed in the same binding site of SC2. Compound YB2 formed six H-bonds with the side chains of Ser146 and Arg216. COX2 contains a 25 Å hydrophobic channel. This channel extends into the core of the catalytic domain [33]. It appears that all of the three compounds have the capability to occupy the upper part of the aforementioned hydrophobic channel between Arg120 and near Tyr385 [34,35]. Compounds of this type can be hopeful COX2 inhibitors [36,37]. The molecular interaction of the most active compounds DB2, SC2 and YB2, with the COX2 enzyme was further validated by fluorescence spectral studies.

### 2.3. Fluorescence Spectral Studies

#### 2.3.1. Fluorescence Quenching Mechanisms

**Figure 4:**
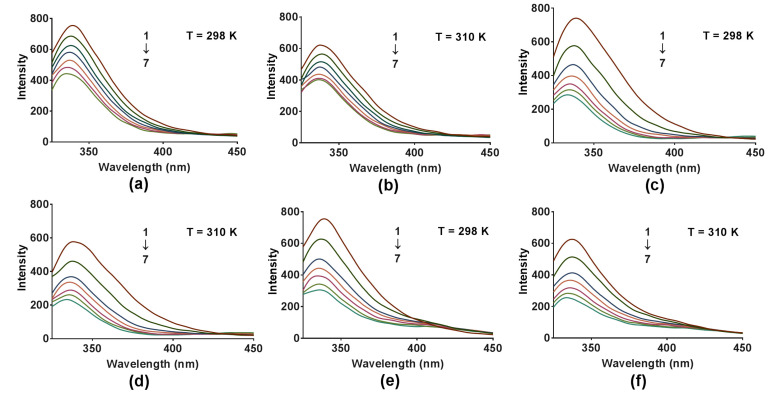
Spectra of COX2 (6.0 × 10^−8^ mol∙L^−1^) fluorescence quenching upon binding to compounds DB2 (**a**,**b**), SC2 (**c**,**d**) or YB2 (**e**,**f**) (1→7: 0, 0.75, 1.50, 2.25, 3.00, 3.75, 4.50 × 10^−5^ mol∙L^−1^) at 298 K and 310 K.

**Figure 5:**
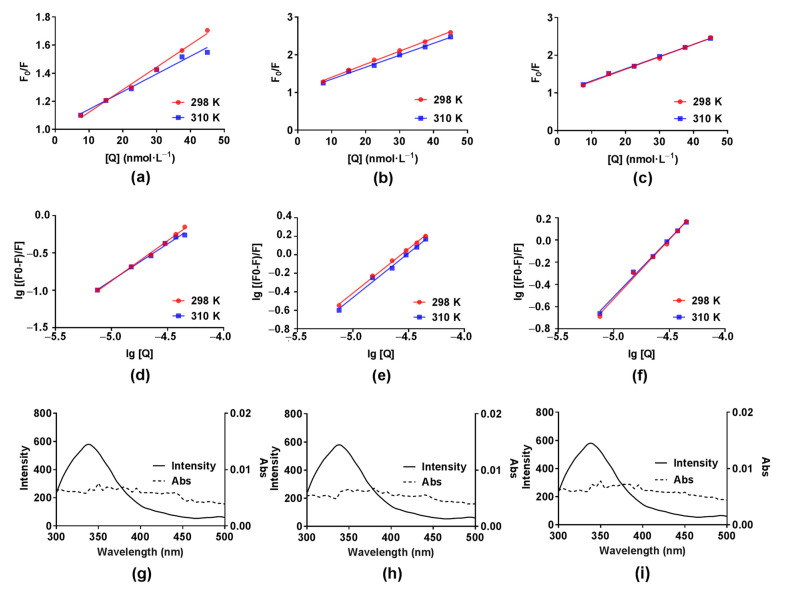
Stern-Volmer plots for COX2-DB2 (**a**), COX2-SC2 (**b**) and COX2-YB2 (**c**) at 298 K and 310 K; Double logarithmic plots for COX2-DB2 (**d**), COX2-SC2 (**e**) and COX2-YB2 (**f**) at 298 K and 310 K; The overlap between COX2 (5.0 × 10^−8^ mol∙L^−1^) fluorescence spectra (solid line) and compounds DB2 (**g**), SC2 (**h**) or YB2 (**i**) (5.0 × 10^−8^ mol∙L^−1^) absorbance spectra (short dot).

The emitted fluorescence spectra of COX2 solution (6.0 × 10^−8^ mol∙L^−1^) without and with an increasing concentration of compounds DB2, SC2 or YB2 (7.5 × 10^−6^ mol∙L^−1^ to 4.5 × 10^−5^ mol∙L^−1^) at different temperatures are shown in Figure 4a,b, Figure 4c,d or Figure 4e,f, respectively. According to Figure 4, the COX2 emission fluorescence intensity gradually decreased with increasing concentration of compounds DB2, SC2 or YB2, which indicates that the three compounds can bind to COX2. The quenching constant *Kq* and Stern–Volmer constant *Ksv* at different temperatures are usually used to identify the quenching mechanisms, static or dynamic. The former is caused by ground-state complex formation, the latter is diffusion [38]. For the static quenching mechanism, the quenching constant decreases with temperature increase [38]. In this part, we calculated the *Kq* and *Ksv* values by using a universally accepted Stern–Volmer equation [38]:

(1) $$\frac{F_{0}}{F}=1+K_{q}\tau_{0}\left[Q\right]=1+K_{sv}\left[Q\right]$$

where *F*_0_ and *F* are the protein fluorescence intensities in the absence and the presence of quencher (compounds DB2, SC2 or YB2), respectively. *Kq* = *Ksv*/$\tau_{0}$. $\tau_{0}$ is the fluorescence lifetime of the fluorophore without the quencher (10^8^ s) [38]. [Q] is the concentration of quencher. The Stern-Volmer plots of COX2 fluorescence quenching by compounds DB2, SC2 or YB2 are presented in Figure 5a, Figure 5b or Figure 5c, respectively. The *K*sv and *K_q_* values for the interaction of the three compounds with COX2 at two different temperatures are listed in Table 1. According to Table 1, the *Ksv* and *K_q_* values for all three systems (COX2-DB2, COX2-SC2 and COX2-YB2) decreased with increasing temperature. Therefore, we considered that the quenching was static quenching. To further verify that the system was static quenching, dynamic quenching was assumed first. Because the average life of the biological macromolecule was seen as 10^8^ s [38], the *Kq* values were more than the maximum diffusion collision quenching constant (2 × 10^10^ L∙mol^−1^∙S^−1^), which indicated that the interaction was due to ground-state complex formation [38].

#### 2.3.2. Binding Constants and Number of Binding Sites

For the static quenching, we can use the double logarithm equation to obtain the binding constant (*K_A_*) and the number of binding sites per protein (*n*) [39]:

(2) $$\mathrm{lg}\frac{F_{0}-F}{F}=\mathrm{lg}K_{A}+n\mathrm{lg}\left[Q\right]$$

The plots of lg [(*F*_0_ − *F*)/*F*] vs. lg [Q] for COX2-DB2, COX2-SC2 and COX2-YB2 at 298 K and 310 K are shown in Figure 5d–f, respectively. As seen in Table 1, the values of *n* were between 1.01 to 1.10 at 298 and 310 K, suggesting that DB2, SC2 or YB2 had one high affinity binding site on COX2. From the intercepts of the curves, we could calculate the values of *K_A_* (Table 1), which were all larger than 10^5^ L∙mol^−1^. Five orders of magnitude *K_A_* value obtained with this method meant considerably great affinities [38,40,41]. That demonstrated that the affinities of compounds DB2, SC2 or YB2 for COX2 were considerably strong. At the same time, the *K_A_* values of DB2, SC2 and YB2 were approximately an order of magnitude larger than those of the control compound (the leading compound, [3-isobutyl-4-methoxy-5 -(pyrrolidine-1-carbonyl)-phenyl]-butyric acid ethyl ester) at 298 K and 310 K, respectively, which further proved that the complexation of DB2, SC2 and YB2 with COX2 were highly favourable. According to Table 1, an increase in temperature from 298 K to 310 K resulted in a decrease in the *K_A_* values. This variation showed that the interaction between the three compounds and COX2 were weakening with increasing temperature. Furthermore, the reduction of the stability and exothermic binding reaction between three compounds and COX2 were suggested. These results are favored by the enthalpy change (Table 2). The obtained *K_A_* values of COX2-DB2 complexes and COX2-YB2 complexes varied rapidly with different temperatures, which meant that the temperature affected the interaction directly.

#### 2.3.3. Thermodynamic Parameters

There are four main kinds of non-covalent forces between drugs and proteins. These are electrostatic force, hydrogen bonding, hydrophobic force and van der Waals force [42,43]. The kind of interaction between compounds DB2, SC2 or YB2 and COX2 can be obtained from thermodynamic parameters, including the free energy change (Δ*G*), enthalpy change (Δ*H*) and entropy change (Δ*S*) of the reaction. The three thermodynamic parameters can be obtained according to the published equations [42,44]:

(3) ΔG=−RTlnK

(4) $$\ln\frac{K_{2}}{K_{1}}=\Delta H\left(\frac{1}{T_{1}}-\frac{1}{T_{2}}\right)\times \frac{1}{R}$$

(5) ΔG=ΔH−TΔS

*K* represents the constant of the binding between protein and quencher at different temperatures. *R* = 8.314 J∙mol^−1^∙K^−1^ [38]. As shown in Table 2, the *ΔG* and Δ*H* values were negative, indicating that the binding processes between compounds DB2, SC2 or YB2 and COX2 were initiative and thermopositive. Besides, the positive value of Δ*S* suggested that binding between compound SC2 and COX2 was mainly hydrophobic-force-driven, which was also shown in COX2-YB2 systems. A negative Δ*H* value was usually seen as proof for hydrogen bonds in the binding process [45,46]. This part corroborated the existence of hydrogen bonding in all three compound-COX2 systems, which agreed with the docking results.

#### 2.3.4. Fluorescence Resonance Energy Transfer Studies

The transfer of energy from the protein donor to the ligand acceptor is generally seen in strong protein–ligand complexes. In addition to divulging the binding activities, assessments of the energy transfer efficiency can reveal the distance between the ligand and the ligand binding site on the protein [47]. According to Förster theory, the efficiency depends not only on the orientation and distance of the molecules but also on the extent of overlap between the donor emission and acceptor absorption. The distance between the donor and acceptor must be in the 2 to 8 nm range [47,48]. The overlaps of fluorescence emission spectrum of COX2 with absorption spectrum of compounds DB2, SC2 or YB2 are presented in Figure 5g–i, respectively. The energy transfer efficiency (*E*) from donor to acceptor was calculated by a widely used method [47,48]:

(6) $$E=\frac{R_{0}^{6}}{R_{0}^{6}+r^{6}}=\frac{F_{0}-F}{F_{0}}$$

(7) $$R_{0}^{6}=8.79\times 10^{-25}\kappa^{2}N^{-4}\varphi J$$

(8) $$J=\frac{\sum^{\;}F\left(\lambda \right)\varepsilon \left(\lambda \right)\lambda^{4}\Delta \lambda }{\sum^{}F\left(\lambda \right)\Delta \lambda }$$

Here, *r* is the separation distance between the donor (COX2) and acceptor (DB2, SC2 or YB2). *R*_0_ is the Förster critical distance when *E* = 50%. *κ*^2^ is the orientation space factor. *N* is the refractive index of medium surrounding the fluorophores. φ is the fluorescence quantum yield of the donor. *J* is overlap integral of the donor fluorescence emission spectrum and acceptor absorption spectrum. *F*(*λ*) is the fluorescence intensity of the protein at the wavelength *λ*, *ε*(*λ*) is the molar absorption coefficient of acceptor at *λ*. Using the values of *κ*^2^ = 2/3, *N* = 1.336 and φ = 0.15 [49], *J* and *r* were calculated (Table 2). The distance between compounds DB2, SC2 or YB2 and the amino acid residue in COX2 is below the upper limit of 8 nm, suggesting a high possibility of energy transfer from COX2 to the three compounds.

#### 2.3.5. Conformational Studies

**Figure 6:**
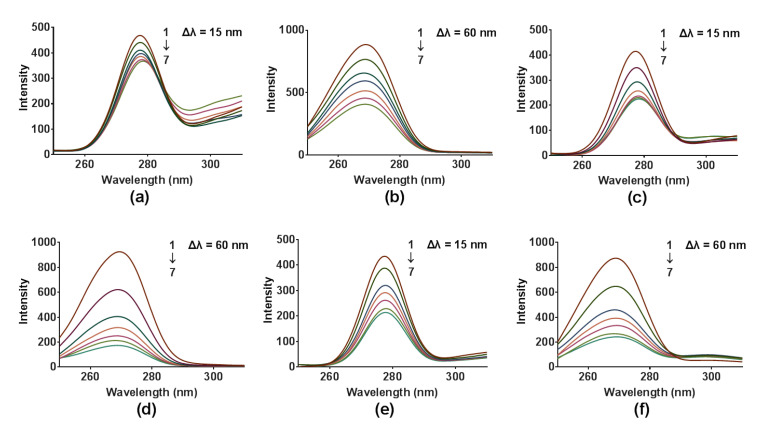
Synchronous fluorescence spectra of COX2 (6.0 × 10^−8^ mol∙L^−1^) in the presence of different concentrations of compounds DB2 (**a**,**b**), SC2 (**c**,**d**) and YB2 (**e**,**f**) (1→7: 0, 0.75, 1.50, 2.25, 3.00, 3.75, 4.50 × 10^−5^ mol∙L^−1^) at 298 K.

To investigate the effects of compounds DB2, SC2 or YB2 on the conformation changes of COX2, synchronous fluorescence and three-dimensional fluorescence spectroscopy were applied. Synchronous fluorescence spectra provide characteristic information about the molecular condition of proteins in the neighborhood of Tyr and Trp [43]. After adding different concentrations of compounds DB2, SC2 or YB2, the synchronous fluorescence spectra of COX2 are displayed in Figure 6a–f, respectively. The maximum wavelengths of Tyr and Trp centered at 277 and 270 nm, respectively, without dramatic shift. The fluorescence intensity of Trp was stronger than that of Tyr. This was an indication that the fluorescence of COX2 was mainly attributed to Trp. Furthermore, the increase in concentration of compound SC2 directly decreased the fluorescence intensity of Tyr and Trp. The positions of spectral peaks were not shifted. This indicated that the interaction between compound SC2 and COX2 changed the microenvironment of Tyr and Trp. Furthermore, the fluorescence intensity of Trp decreased more steeply than that of Tyr. This indicated that Trp gave more contributions to this binding than Tyr did. These data suggested that Trp might may be nearer to binding sites than Tyr did. Similarly, COX2-DB2 and COX2-YB2 exhibited the same results. This indicated that binding of these three compounds could cause the conformation changes of COX2.

**Figure 7:**
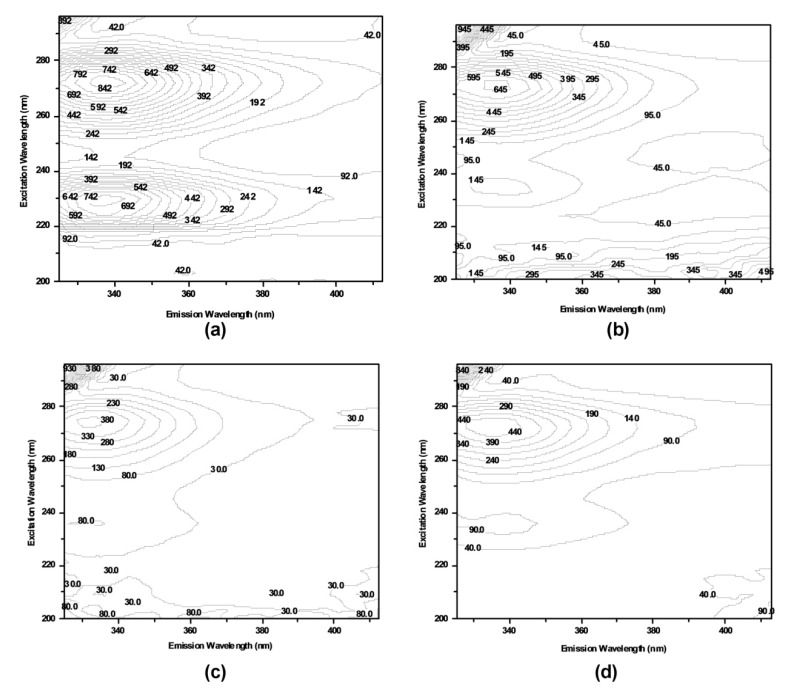
Three-dimensional fluorescence contour spectra of COX2 (5.0 × 10^−8^ mol∙L^−1^) in the absence (**a**) and presence of compounds DB2 (**b**), SC2 (**c**) or YB2 (**d**) (4.50 × 10^−5^ mol∙L^−1^) at 298 K.

Three-dimensional fluorescence spectroscopy can reflect the microenvironment of related fluorophores. It was employed to study the structural changes of COX2 before and after binding [50]. The three-dimensional fluorescence spectra of COX2, COX2-SC2, COX2-DB2 and COX2-YB2 are presented in Figure 7a–d, respectively. Peak 1 and peak 2 were maintained at 272/338 nm and 230/337 nm, respectively. Peak 1 revealed the intrinsic fluorescence information of Trp and Tyr residues of COX2 due to pi-to-pi* transition related to the tertiary structure changes. Peak 2 reflected the fluorescence information of the polypeptide backbone structure of COX2, involving pi-to-pi* transition related to the secondary structure changes [42,50]. According to Figure 7 and Table 3, both peak 1 and peak 2 of COX2 had been quenched. The results indicated that compound SC2 had formed a complex with COX2 at ground state. The protein conformational alteration was subsequently induced. Furthermore, the fluorescence intensity of peak 1 was higher than that of peak 2 in the spectra for COX2-SC2 complex (Table 3). It implies that the fluorescence quenching of COX2 by compound SC2 on peak 2 was more evident than on peak 1. We could draw a conclusion that the interaction of compound SC2 with COX2 induced the slight opening of the polypeptides of protein, thereafter changing the conformation of COX2. The exposure of some hydrophobic regions, which were previously buried, was subsequently increased [42]. Comparison of Figure 7a with Figure 7c also implied that there was a blue shift of 5.5 nm for peak 1, and 4 nm for peak 2. This revealed that compound SC2 binding induced microenvironmental changes in amino acid residues in COX2. These changes induced the increase in hydrophobicity of COX2, decrease in collisions between COX2 molecules and water, and increase in the quantum yield of COX2. COX2-DB2 and COX2-YB2 showed the same results. These results were consistent with the fluorescence quenching assay, FRT study and synchronous fluorescence measurement. Taken together, these combined results implicate the ability of compounds DB2, SC2 and YB2 to bind to COX2.

### 2.4. Biological Activities

#### 2.4.1. In Vitro Antioxidant Activity

**Figure 8:**
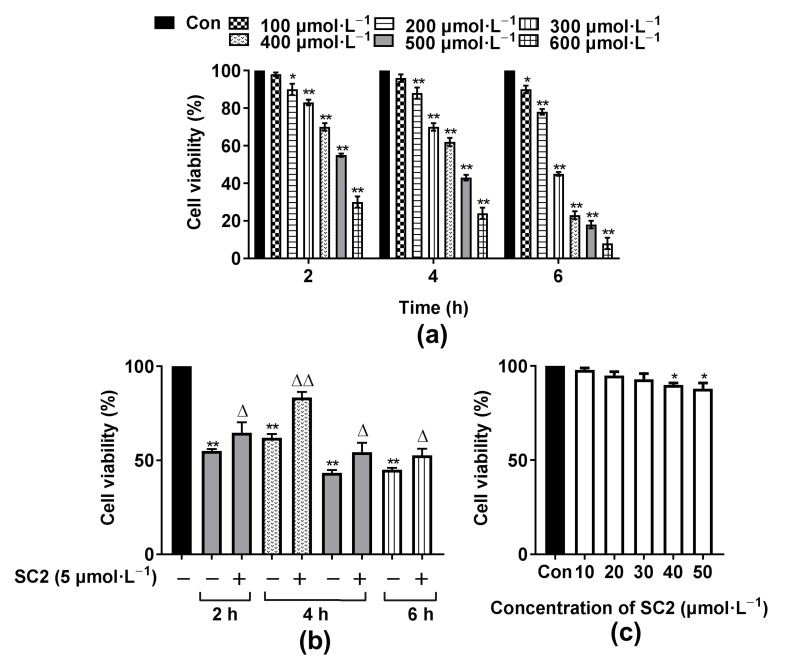
(**a**) Cell viability after exposure to 100, 200, 300, 400, 500 or 600 µmol∙L^−1^ H_2_O_2_ for different durations; (**b**) Cell viability after exposure to 5 µmol∙L^−1^ SC2 and various concentrations of H_2_O_2_ for different durations; (**c**) Cytotoxocity of compound SC2 (compared with the control group, * *p* < 0.05, ** *p* < 0.01; compared with the model group, ^△^ *p* < 0.05, ^△△^ *p* < 0.01).

Hydrogen peroxide (H_2_O_2_), a small molecule and functions as oxidative stress and a second messenger which contributes to cell damage or death, could directly induce inflammation in several cell lines. H_2_O_2_-induced cell damage is a commonly used model for antioxidant activity evaluation [51,52]. The viability of EA.hy926 cells treated with different concentrations of H_2_O_2_ at different times are shown in Figure 8a. As shown in Figure 8a, with increasing concentrations of H_2_O_2_, cell viability decreased in a dose-dependent and time-dependent manner. H_2_O_2_ exposure caused 50% cell viability loss was widely selected [51,53,54,55,56]. Thus, the cell viability of 43 ± 1.5% (500 µmol·L^−1^ H_2_O_2_ at 4 h), 45 ± 1.0% (300 µmol·L^−1^ at 6 h), 55 ± 0.9% (500 µmol·L^−1^ at 2 h) and 62 ± 2.2% (400 µmol·L^−1^ at 4 h) were chosen for further screening. According to Figure 8b, treatment with 5 µmol·L^−1^ of compound SC2 protects the cells from H_2_O_2_ induced cell damage as compared with four pre-chosen H_2_O_2_ treatments. The most efficient cytoprotective effect of SC2 was observed when the H_2_O_2_ exposure time was 4 h and the concentration was 400 μmol·L^−1^. Thus, the concentration of 400 µmol·L^−1^ at 4 h was chosen as the model.

As seen in Table 4, there was no significant difference in the protection rate between six compounds (DC2, SB1, SB2, SC1, YB2, YC2) and the positive controls (fenofibrate and quereetin). Futhermore, SC2 possessed the highest protection rate (90.0 ± 0.054%), which was significantly higher than that of quereetin (*p* < 0.01). Its concentration for the 50% of maximal effect (EC_50_) value was only 6.25 ± 0.415 nM, which was 1000 times greater than that of the positive controls, fenofibrate and quereetin.

#### 2.4.2. Cytotoxocity of Compound SC2

The cytotoxicity of compound SC2 on EA.hy926 cells was detected by 3-(4,5-dimethyl-2-thiazolyl)-2,5-diphenyl-2-H-tetrazolium bromide (MTT) assay. As shown in Figure 8c, the cell survival rate between the groups with concentrations of SC2 less than 30 µmol·L^−1^ and the control group were not significantly different. Thus, in subsequent cell-based experiments, the concentration of SC2 used was less than 10 µmol·L^−1^, which was harmless to cells.

#### 2.4.3. Effects of Oxidative Stress-Related Factors

**Figure 9:**
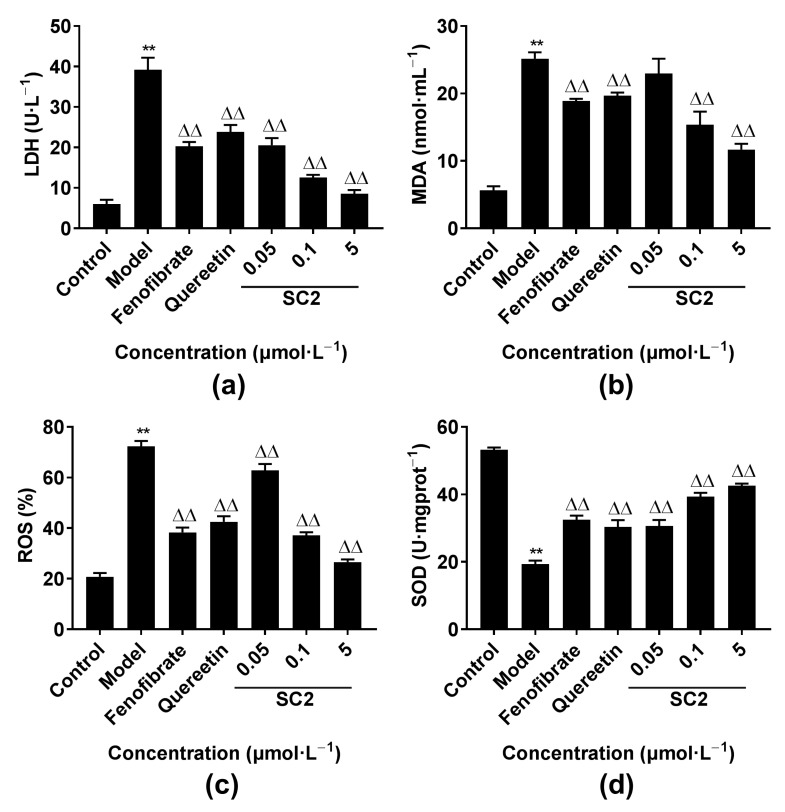
Improved biochemical indicators in compound SC2-treated group caused by oxidative stress damage. (**a**) Lactate dehydrogenase (LDH) content; (**b**) Malondialdehyde (MDA) content; (**c**) Reactive oxygen species (ROS) content; (**d**) Superoxide dismutase (SOD) activity (compared with that in the control group, ** *p* < 0.01; compared with that in the model group, ^△△^ *p* < 0.01).

In the course of oxidative stress, the balance between oxidation and antioxidant activity of cells was disturbed, and the levels of ROS, malondialdehyde (MDA) and lactate dehydrogenase (LDH) increased, while the activity of superoxide dismutase (SOD) enzyme in cells decreased. As shown in Figure 9, compared with that in the control group, the levels of ROS, MDA and LDH in the model group significantly increased (*p* < 0.01), and the activity of SOD was significantly reduced (*p* < 0.01). However, the levels of ROS, MDA and LDH in the SC2 group were significantly reduced (*p* < 0.01), and the activity of SOD significantly increased (*p* < 0.01) compared with that in the model group. These results showed that SC2 could effectively reduce oxidative stress and enhance the antioxidant defense system during oxidative stress damage.

## 3. Discussion

We have investigated the interaction of newly synthesised double hydroxide-based benzophenone derivatives with the COX2 enzyme, a classical therapeutic target in many inflammation and oxidative stress associated diseases. COXs present in two functional isoforms COX1 and COX2 [1,2]. The constitutive form of COXs, COX1, is expressed in most cells. COX1 acts as a ‘‘house-keeper” enzyme as it is involved in the maintenance functions of internal organs [3]. However, some serious complications of NSAIDs are attributed to the inhibition of COX1 and restrict the clinical application. Compounds binding with constitutively expressed COX1 will affect the normal function of cells and induce side effects. So, attention has been given to identify the binding ability of small molecules with COX2 instead of COX1 in drug discovery, even though COX1 possesses a quite similar binding pocket with COX2. 

A BLI experiment was performed first. This technique offered unique advantages in that it can be rapidly operated and requires smaller quantities of both protein and ligand compared with conventional analytical techniques. It has been recently recommended as a fast and cost-effective tool to screen and optimize ligands to specific targets [57]. Our BLI analysis suggested that three of twelve tested compounds (DB2, SC2 and YB2) interacted with the protein and provided the kinetic parameters. The best putative binding mode was elucidated by molecular docking studies. Docking results revealed that compounds DB2, SC2 and YB2 showed hydrogen bonding interaction with amino acids Ser146 and Arg216 indicating that three tested compounds shared a relatively similar binding mode. The polyhydroxy benzophenone moiety of the three tested compounds was trapped in the hydrophobic channel, where it increased the binding affinity. Subsequently, we performed multi-spectroscopy studies. The interactions between compounds DB2, SC2 or YB2 and COX2 were confirmed as being due to ground-state complex formation. The processes were initiative and reversible. All three compounds formed hydrogen bonds with COX2, which agreed with the docking results. Hydrophobic force was the main intermolecular force in COX2-SC2 and COX2-YB2 systems. The synchronous fluorescence studies demonstrated that Trp residues of COX2 came into contact with the binding sites closer to the Tyr residues. The FRET analysis further suggested that the distance between compounds DB2, SC2 or YB2 and the amino acid residue in COX2 was below 8 nm. 

Together, the above joint computational and experimental results suggested that among all the twelve compounds, DB2, SC2 and YB2 could successfully bind with COX2. The order of binding constants (*K_D_* and *K_A_*) obtained from BLI and multi-spectral methods showed different trends due to certain inadequacies. However, the binding constants obtained from the same methods were of the same order of magnitude indicating that compounds DB2, SC2 and YB2 could bind COX2 more firmly than the remaining compounds. 

As shown in Figure 1, compounds DB2, SC2 and YB2 all possessed a phenolic hydroxyl group at C-2’. Removal of the hydroxyl group at the C-2’ position resulted in no COX2 binding activity as compared to these three compounds. The higher COX2 binding activity of these three compounds may be attributed to the presence of three phenolic hydroxyl groups adjacent to each other on the B ring. The number of hydroxyl groups in a molecule shows the potent hydrogen donor features due to electron delocalization over the molecule, thus stabilizing free radicals [58,59]. It has been indicated that the hydroxyl group is crucial in contributing to the enhanced COX2 inhibitory activity effects of benzophenone derivatives [60,61]. Another important structural feature is probably the planarity of the system. Planarity permits conjugation between the carbonyl group and hydroxyl group in para-position, thus raising the hydrogen donation power due to delocalization of the radicals formed. In solution, this would possibly result in keto–enol tautomerism, as reported for analogical double hydroxide-based benzophenones [59]. Keto–enol tautomer of such kind is classically rationalized depending on the electronic resonance effect caused by one of the phenolic hydroxyl groups, which would make the inhibitor attach to enzymes firmly [62]. Both features come into play in the case of compounds DB2, SC2 and YB2, which rationalizes their high COX2 binding activities [59].

Oxidative stress and inflammation are closely connected with each other, involving complicated feedforward and feedback loops. Increased levels of ROS have been found in many inflammation associated diseases [34,63]. Thus, the use of a single agent targeting COX2 enzyme along with oxidative stress process would probably be favorable. Significantly increased antioxidant activity against H_2_O_2_-induced cell damage was observed in all the groups treated with twelve tested derivatives. In particular, compound SC2 showed the strongest activity. Its EC_50_ value was three orders of magnitude higher than that of fenofibrate and quereetin, two powerful and known compounds with antioxidant property. Additionally, SC2 caused certain improvements in serum levels of biochemical indicators (ROS, MDA, LDH and SOD) in H_2_O_2_-induced cell model.

## 4. Materials and Methods

A Fortebio Octet System (FortéBio, San Francisco, CA, USA) and fluorescence spectrophotometer (LS55, Perkin-Elmer, Waltham, MA, USA) were used to measure the binding kinetics of the target compounds to COX2. The UV-Vis absorption spectrum was recorded on a UV-1800PC spectrophotometer (MAPADA, Shanghai, China). Nickel-charged tris-NTA (Ni-NTA) biosensors were purchased from FortéBio Pall life sciences (San Francisco, CA, USA). 

The human umbilical vein endothelial cell line (EA.hy926) was purchased from Shanghai Zhongzhou Biotechnology Co. (Shanghai, China). Dulbecco’s modified Eagle medium (DMEM) was obtained from Boster Biological technology Co. (Wuhan, China). Cellmax fetal bovine serum (FBS) was obtained from Minhai Bioengineering Co. (Lanzhou, China). MTT was obtained from Beijing Solarbio Science & Technology Co. (Beijing, China). The assay kits for LDH, MDA, ROS and SOD were purchased from Nanjing Jiancheng Bioengineering Institute (Nanjing, China). Recombinant active human COX2 was purchased from Sino Biological Co. (Beijing, China). Ultrapure water was prepared by a Milli-Q water purification system (Millipore, Billerica, MA, USA). All other reagents and solvents were of analytical grade.

### 4.1. Biolayer Interferometry Studies

His-tagged COX2 solution (40 μg∙mL^−1^), which was prepared in phosphate buffer saline (PBS), was immobilized on Ni-NTA biosensor tips for 600 s. To remove drift and well-to-well artifacts, we performed double reference subtraction with FortéBio data analysis software. All the twelve compounds stock solutions (10 mM) were prepared in dimethyl sulfoxide (DMSO). The stock solutions were diluted with PBS containing 0.1% between 20 (PBST) to 100 μM for the initial assay. Typically, for re-assay, the stock solutions were serially diluted (4–6 point serial dilutions) in PBST. Compound testing was performed sequentially taking baseline, association and dissociation steps. Each of these steps took 60 s. The alignment of data was carried out using baseline signal and curves fitted with a one to one best-fit model in FortéBio’s data analysis software.

### 4.2. Molecular Docking

Molecular docking was performed using the Dock-ligands program in Sybyl X-2.0 version (Tripos, St. Louis, MO, USA). Molecular structures of COX2 (PDB code 5f1a) used for molecular docking were obtained from the RSCB database (http://www.rcsb.org/pdb, accessed on 23 April 2021). In order to prepare the COX2 structures, all the water molecules and ligands were removed. Additionally, hydrogen atoms were added. The twelve compounds were constructed using the Sybyl Sketcher model and conformational minimizations were performed with the Powell method (Tripos force field and Gasteiger–Huckel charge modification) to the lowest energy conformers. The number of interactions was optimized 10,000 times. Unless otherwise specified, the selected parameters were defaults.

### 4.3. Fluorescence Spectrum Measurement

The fluorescence intensity of the system was measured with a 1.0 cm quartz cell. COX2 protein was dissolved in PBS for a final concentration of 6.0 × 10^−8^ mol∙L^−1^. DB2, SC2 or YB2 stock solutions were prepared in singleton at 1.5 × 10^−3^ mol∙L^−1^ in PBS containing 0.1% DMSO. 2.0 mL COX2 solution was separately titrated by continuing additions of 10 μL compounds DB2, SC2 or YB2 solution). After incubation at 298 K or 310 K, fluorescence quenching spectra were recorded in the region 325-450 nm at a scan rate of 1200 nm∙min^−1^. The local environment of Tyr and Trp residues was quite sensitive at 280 nm, so the excitation wavelength was set at this wavelength. Typically, 280 nm is also used to measure the changes in protein tertiary structure [44]. The slit widths of emission and excitation were both fixed at 10 nm. To observe spectra behaviors of Tyr and Trp groups of COX2, the wavelength interval between the excitation and emission wavelengths (Δλ) was set as 15 nm and 60 nm, respectively. Three-dimensional fluorescence spectroscopy was obtained for an excitation range of 200–300 nm and an emission range of 325–400 nm. Appropriate buffer solutions, determined under the same operation conditions, were used as blanks. Each spectrum was recorded three times.

### 4.4. Pharmacological/Biological Assays

#### 4.4.1. Cell Lines and Cell Culture

The human EA.hy926 cells were cultured in DMEM supplemented with 10% heat-inactivated FBS, 100 U·mL^−1^ penicillin, and 100 μg·mL^−1^ streptomycin under 5% CO_2_ at 37 °C. All cells used for the experiment were in a logarithmic growth phase according to cell growth.

#### 4.4.2. Establishment of the Oxidative Damage Model

Compound SC2 stock solutions were prepared in DMSO. The stock solutions were diluted with FBS free DMEM to different target concentrations (DMSO concentrations ≤ 0.5% *v*:*v*). Cells (100 μL, 2 × 10^5^ cells∙mL^−1^) were seeded on 96-well plates and cultured for 24 h. The medium was then replaced with fresh media containing H_2_O_2_ with different concentrations (0, 100, 200, 300, 400, 500, and 600 μmol·L^−1^ in FBS free DMEM) and compound SC2 (50, 40, 30, 20, 10 µmol·L^−1^), which were transferred to the incubator and cultured for additional time (2, 4, and 6 h). The medium was then replaced with 100 μL of FBS free DMEM and MTT (10 μL, 5 mg·mL^−1^ in PBS) and after 4 h the medium was replaced with DMSO (100 μL). A microplate reader (SpectraMaxPlus384, Molecular Devices, United States) was used to record the absorbance at 490 nm for each well of the plates. Cell viability was measured via MTT assay. Survival rate (%) = (A_sample group_/A_control group_) × 100%.

#### 4.4.3. Protective Effect of Target Compounds on Oxidative Damage

All the twelve compounds and positive controls, fenofibrate and quercetin, shared the same preparation method as mentioned in Section 4.4.2. Cells (100 μL, 2 × 10^5^ cells·mL^−1^) were seeded in 96-well plates for 24 h. Cells were treated with different groups and cell viability was measured via MTT assay. The protection rates to oxidative damage were determined and EC_50_ values were calculated. The protection rates to cells were calculated using the following formula: Protection rate (%) = (A_pretreatment group_ − A_model group_)/(A_control group_ − A_model group_) × 100%.

#### 4.4.4. Activity Assessment of LDH, MDA, SOD and ROS In Vitro

Cells were dispensed into 24-well plate wells for 24 h and pretreated with target compound SC2 (5, 0.1, 0.05 µmol·L^−1^) or the positive controls (10 µmol·L^−1^) for 24 h, followed by exposure to 400 μmol·L^−1^ H_2_O_2_ for 4 h. The cells were collected and the activities of LDH, MDA, SOD and ROS were determined according to the kits’ instructions.

### 4.5. Statistical Analysis

Statistical analyses were performed using a one-way analysis of variance (ANOVA) and a Dunnett post hoc test. All data are expressed as the mean ± standard deviation. Statistical analyses were performed using Graphpad Prism 7. *p* < 0.05 were considered significant.

## 5. Conclusions

This study provided further molecular details for the generation of novel COX2 inhibitors. The best combined profile of COX2 binding and antioxidant activity was displayed by the novel double hydroxide-based benzophenone derivative, SC2. Further studies should investigate whether SC2 can act as COX2 inhibitor in vivo in order to develop it as a potent candidate.

## Figures and Tables

**Table 1:** The Stern-Volmer quenching constant (*Ksv*), bimolecular quenching constant (*Kq*) and binding constant (*K_A_*) for the interaction of compounds DB2, SC2 and YB2 with COX2 at 298 K and 310 K.

Compound	T (K)	*Ksv* (×10^4^ L∙mol^−1^)	*Kq* (×10^12^ L∙mol^−1^∙S^−1^)	R^a^	*K_A_* (L∙mol^−1^)	*n*	R^b^
DB2	298	1.43 ± 0.188	1.43 ± 0.188	0.994	35,432 ± 4052.5 **	1.10 ± 0.0208	0.994
	310	1.14 ± 0.380	1.14 ± 0.380	0.972	13,677 ± 2797.7 *	1.03 ± 0.0633	0.986
SC2	298	2.31 ± 0.988	2.31 ± 0.988	0.991	23,955 ± 2224.7 **	1.01 ± 0.0469	0.996
	310	2.07 ± 1.03	2.07 ± 1.03	0.978	21,881 ± 4951.0 **	1.02 ± 0.0731	0.987
YB2	298	3.49 ± 1.69	3.49 ± 1.69	0.995	63,827 ± 2612.1 **	1.07 ± 0.0551	0.993
	310	2.58 ± 0.562	2.58 ± 0.562	0.995	46,411 ± 5519.0 **	1.07 ± 0.0306	0.995
Control compound	298	1.71 ± 0.297	1.71 ± 0.297	0.990	4436.3 ± 718.24	0.947 ± 0.116	0.982
	310	1.05 ± 0.157	1.05 ± 0.157	0.981	1417.0 ± 92.261	0.785 ± 0.0195	0.992

R^a^ is the correlation coefficient for the *K*_SV_ and *Kq* values; R^b^ is the correlation coefficient for the *K_A_* values; Compared with the control compound, * *p* < 0.05, ** *p* < 0.01.

**Table 2:** Thermodynamic parameters, the effective spectral overlap (*J*) and the separation distance (*r*) for the interaction of compounds DB2, SC2 and YB2 with COX2 at 298 K and 310 K.

Compound	T (K)	Δ*H* (kJ∙mol^−1^)	Δ*S* (J∙K^−1^)	Δ*G* (kJ∙mol^−1^)	*J* (×10^−13^ cm^3^∙L∙mol^−1^)	*r* (nm)
DB2	298	−61.6 ± 8.20	−120 ± 28.1	−25.9 ± 0.280	1.64	6.42
	310			−24.6 ± 0.561		
SC2	298	−6.74 ± 9.11	61.2 ± 31.3	−25.0 ± 0.229	1.55	6.62
	310			−25.7 ± 0.599		
YB2	298	−20.7 ± 6.48	22.6 ± 21.9	−27.4 ± 0.101	1.72	6.47
	310			−27.7 ± 0.318		

**Table 3:** The characteristic parameters of three-dimensional fluorescence spectra.

System	Peak	Peak Position λex/λem (nm/nm)	Relative Intensity (I)
COX2	1	272/338	874
	2	230/337	775
COX2-DB2	1	272/336.5	665
	2	236/337	153
COX2-SC2	1	275/332.5	395
	2	236/333	82.9
COX2-YB2	1	272/334.5	472
	2	236/336	103

**Table 4:** Protective rate of target compounds.

Compound	Protective Rate (%) ^a,b^	EC_50_ (nM) ^a,b^
DB1	52.70 ± 2.021 ^##,^ *	7080 ± 92.56
DB2	59.12 ± 5.571 ^##^	1360 ± 463.4
DC1	60.30 ± 7.399 ^##^	3310 ± 242.5
DC2	67.34 ±6.136	1550 ± 924.4
SB1	75.83 ±7.060	12.01 ± 4.805
SB2	76.91 ± 7.558	25.22 ± 12.16
SC1	80.62 ± 3.605	1910 ± 521.3
SC2	90.02 ± 5.410 **	6.254 ± 0.4153
YB1	54.73 ± 1.900 ^##,^ *	8510 ± 956.8
YB2	69.32 ± 6.164	417.5 ± 24.56
YC1	55.20 ± 3.665 ^##^	1910 ± 823.4
YC2	67.01 ± 7.390	3850 ± 925.3
Fenofibrate	78.22 ± 9.861	6920 ± 610.2
Quereetin	69.53 ± 3.284	7980 ± 1230

^a^ Average value obtained from three independent experimental measurements; ^b^ Mean ± standard deviation (*n* = 3); Compared with the fenofibrate treated group, ^##^ *p* < 0.01; Compared with the quereetin treated group, * *p* < 0.05, ** *p* < 0.01.

## Data Availability

Not applicable.

## Sample Availability

Samples of the compounds are not available from the authors.
